# Supplementary material for: Does a degree in medicine or a specialist programme or socioeconomic status advance the career of general practitioners in primary healthcare?
Source: PLoS One. 2026 Mar 30;21(3):e0346026. doi: 10.1371/journal.pone.0346026 (PMC13035118; doi:10.1371/journal.pone.0346026)
Supplement: S1_File — (PDF) [file pone.0346026.s001.pdf]

Regression

Descriptive Statistics

	Mean	Std. Deviation	N
RE	.5280	.53558	858
Gender	1.3951	.48916	858
Ethni	1.8450	.36213	858
Age	27.3695	1.11418	858
RL	1.6270	.48387	858
GPtype2	.1981	.39883	858
GPtype3	.1807	.38495	858
GPtype4	.1375	.34461	858
GPtype5	.1515	.35876	858

Correlations

		RE	Gender	Ethni	Age	RL	GPtype2
Pearson Correlation	RE	1.000	.018	-.023	-.012	-.005	-.141
	Gender	.018	1.000	.017	.034	.032	-.061
	Ethni	-.023	.017	1.000	.050	.169	.140
	Age	-.012	.034	.050	1.000	.018	-.055
	RL	-.005	.032	.169	.018	1.000	-.064
	GPtype2	-.141	-.061	.140	-.055	-.064	1.000
	GPtype3	-.050	-.132	-.159	.002	.130	-.233
	GPtype4	-.015	.037	-.007	.041	.035	-.198
	GPtype5	-.052	.051	.037	.055	-.010	-.210
Sig. (1-tailed)	RE	.	.300	.253	.358	.445	.000
	Gender	.300	.	.312	.162	.177	.038
	Ethni	.253	.312	.	.073	.000	.000
	Age	.358	.162	.073	.	.301	.055
	RL	.445	.177	.000	.301	.	.030
	GPtype2	.000	.038	.000	.055	.030	.
	GPtype3	.072	.000	.000	.477	.000	.000
	GPtype4	.335	.138	.423	.117	.153	.000
	GPtype5	.062	.069	.138	.053	.383	.000
N	RE	858	858	858	858	858	858
	Gender	858	858	858	858	858	858
	Ethni	858	858	858	858	858	858
	Age	858	858	858	858	858	858
	RL	858	858	858	858	858	858
	GPtype2	858	858	858	858	858	858
	GPtype3	858	858	858	858	858	858
	GPtype4	858	858	858	858	858	858
	GPtype5	858	858	858	858	858	858

Correlations

		GPtype3	GPtype4	GPtype5
Pearson Correlation	RE	-.050	-.015	-.052
	Gender	-.132	.037	.051
	Ethni	-.159	-.007	.037
	Age	.002	.041	.055
	RL	.130	.035	-.010
	GPtype2	-.233	-.198	-.210
	GPtype3	1.000	-.188	-.198
	GPtype4	-.188	1.000	-.169
	GPtype5	-.198	-.169	1.000
Sig. (1-tailed)	RE	.072	.335	.062
	Gender	.000	.138	.069
	Ethni	.000	.423	.138
	Age	.477	.117	.053
	RL	.000	.153	.383
	GPtype2	.000	.000	.000
	GPtype3	.	.000	.000
	GPtype4	.000	.	.000
	GPtype5	.000	.000	.
N	RE	858	858	858
	Gender	858	858	858
	Ethni	858	858	858
	Age	858	858	858
	RL	858	858	858
	GPtype2	858	858	858
	GPtype3	858	858	858
	GPtype4	858	858	858
	GPtype5	858	858	858

Variables Entered/Removed[a]

Model	Variables Entered	Variables Removed	Method
1	GPtype5, RL, Age, Gender, Ethni, GPtype4, GPtype2, ...[b]	.	Enter

a. Dependent Variable: RE

b. All requested variables entered.

Model Summary

Model	R	R Square	Adjusted R Square	Std. Error of the Estimate	Change Statistics	
					R Square Change	F Change
1	.224[a]	.050	.041	.52439	.050	5.622

Model Summary

Model	Change Statistics		
	df1	df2	Sig. F Change
1	8	849	.000

a. Predictors: (Constant), GPtype5, RL, Age, Gender, Ethni, GPtype4, GPtype2, GPtype3

ANOVA[a]

Model		Sum of Squares	df	Mean Square	F	Sig.
1	Regression	12.367	8	1.546	5.622	.000[b]
	Residual	233.462	849	.275		
	Total	245.829	857			

a. Dependent Variable: RE

b. Predictors: (Constant), GPtype5, RL, Age, Gender, Ethni, GPtype4, GPtype2, GPtype3

Coefficients[a]

Model		Unstandardized Coefficients		Standardized Coefficients	t	Sig.
		B	Std. Error	Beta		
1	(Constant)	.861	.451		1.911	.056
	Gender	-.005	.037	-.005	-.139	.889
	Ethni	-.016	.052	-.011	-.316	.752
	Age	-.005	.016	-.011	-.331	.741
	RL	.006	.038	.006	.169	.866
	GPtype2	-.310	.051	-.231	-6.044	.000
	GPtype3	-.221	.054	-.159	-4.095	.000
	GPtype4	-.179	.058	-.115	-3.111	.002
	GPtype5	-.225	.056	-.151	-4.042	.000

Coefficients[a]

Model		95.0% Confidence Interval for B Lower Bound	Upper Bound	Collinearity Statistics Tolerance	VIF
1	(Constant)	-.023	1.746		
	Gender	-.078	.068	.971	1.030
	Ethni	-.117	.085	.921	1.085
	Age	-.037	.026	.989	1.011
	RL	-.069	.082	.937	1.067
	GPtype2	-.411	-.210	.765	1.308
	GPtype3	-.326	-.115	.746	1.341
	GPtype4	-.292	-.066	.815	1.227
	GPtype5	-.334	-.116	.806	1.241

a. Dependent Variable: RE

Collinearity Diagnostics[a]

Model	Dimension	Eigenvalue	Condition Index	Variance Proportions (Constant)	Gender	Ethni	Age
1	1	5.524	1.000	.00	.00	.00	.00
	2	1.004	2.346	.00	.00	.00	.00
	3	1.001	2.349	.00	.00	.00	.00
	4	1.000	2.350	.00	.00	.00	.00
	5	.301	4.285	.00	.03	.00	.00
	6	.093	7.724	.00	.78	.02	.00
	7	.055	10.036	.00	.13	.11	.00
	8	.023	15.614	.01	.06	.87	.02
	9	.001	82.224	.98	.00	.00	.98

Collinearity Diagnostics[a]

Model	Dimension	Variance Proportions RL	GPtype2	GPtype3	GPtype4	GPtype5
1	1	.00	.00	.00	.00	.00
	2	.00	.10	.43	.01	.08
	3	.00	.32	.01	.19	.13
	4	.00	.01	.01	.36	.31
	5	.00	.51	.44	.43	.46
	6	.19	.03	.06	.01	.01
	7	.80	.03	.00	.00	.01
	8	.00	.00	.05	.01	.00
	9	.00	.00	.00	.00	.00

a. Dependent Variable: RE

Regression

Descriptive Statistics

	Mean	Std. Deviation	N
Promo	.7331	.58159	858
Gender	1.3951	.48916	858
Ethni	1.8450	.36213	858
Age	27.3695	1.11418	858
RL	1.6270	.48387	858
GPtype2	.1981	.39883	858
GPtype3	.1807	.38495	858
GPtype4	.1375	.34461	858
GPtype5	.1515	.35876	858

Correlations

		Promo	Gender	Ethni	Age	RL	GPtype2
Pearson Correlation	Promo	1.000	.006	.047	.075	.048	.052
	Gender	.006	1.000	.017	.034	.032	-.061
	Ethni	.047	.017	1.000	.050	.169	.140
	Age	.075	.034	.050	1.000	.018	-.055
	RL	.048	.032	.169	.018	1.000	-.064
	GPtype2	.052	-.061	.140	-.055	-.064	1.000
	GPtype3	.106	-.132	-.159	.002	.130	-.233
	GPtype4	.131	.037	-.007	.041	.035	-.198
	GPtype5	.317	.051	.037	.055	-.010	-.210
Sig. (1-tailed)	Promo	.	.430	.084	.014	.080	.063
	Gender	.430	.	.312	.162	.177	.038
	Ethni	.084	.312	.	.073	.000	.000
	Age	.014	.162	.073	.	.301	.055
	RL	.080	.177	.000	.301	.	.030
	GPtype2	.063	.038	.000	.055	.030	.
	GPtype3	.001	.000	.000	.477	.000	.000
	GPtype4	.000	.138	.423	.117	.153	.000
	GPtype5	.000	.069	.138	.053	.383	.000
N	Promo	858	858	858	858	858	858
	Gender	858	858	858	858	858	858
	Ethni	858	858	858	858	858	858
	Age	858	858	858	858	858	858
	RL	858	858	858	858	858	858
	GPtype2	858	858	858	858	858	858
	GPtype3	858	858	858	858	858	858
	GPtype4	858	858	858	858	858	858
	GPtype5	858	858	858	858	858	858

Correlations

		GPtype3	GPtype4	GPtype5
Pearson Correlation	Promo	.106	.131	.317
	Gender	-.132	.037	.051
	Ethni	-.159	-.007	.037
	Age	.002	.041	.055
	RL	.130	.035	-.010
	GPtype2	-.233	-.198	-.210
	GPtype3	1.000	-.188	-.198
	GPtype4	-.188	1.000	-.169
	GPtype5	-.198	-.169	1.000
Sig. (1-tailed)	Promo	.001	.000	.000
	Gender	.000	.138	.069
	Ethni	.000	.423	.138
	Age	.477	.117	.053
	RL	.000	.153	.383
	GPtype2	.000	.000	.000
	GPtype3	.	.000	.000
	GPtype4	.000	.	.000
	GPtype5	.000	.000	.
N	Promo	858	858	858
	Gender	858	858	858
	Ethni	858	858	858
	Age	858	858	858
	RL	858	858	858
	GPtype2	858	858	858
	GPtype3	858	858	858
	GPtype4	858	858	858
	GPtype5	858	858	858

Variables Entered/Removed[a]

Model	Variables Entered	Variables Removed	Method
1	GPtype5, RL, Age, Gender, Ethni, GPtype4, GPtype2, ...[b]	.	Enter

a. Dependent Variable: Promo

b. All requested variables entered.

Model Summary

Model	R	R Square	Adjusted R Square	Std. Error of the Estimate	Change Statistics: R Square Change	Change Statistics: F Change
1	.513[a]	.263	.256	.50172	.263	37.825

Model Summary

Model	Change Statistics: df1	Change Statistics: df2	Change Statistics: Sig. F Change
1	8	849	.000

a. Predictors: (Constant), GPtype5, RL, Age, Gender, Ethni, GPtype4, GPtype2, GPtype3

ANOVA[a]

Model		Sum of Squares	df	Mean Square	F	Sig.
1	Regression	76.170	8	9.521	37.825	.000[b]
	Residual	213.710	849	.252		
	Total	289.880	857			

a. Dependent Variable: Promo

b. Predictors: (Constant), GPtype5, RL, Age, Gender, Ethni, GPtype4, GPtype2, GPtype3

Coefficients[a]

Model		Unstandardized Coefficients: B	Unstandardized Coefficients: Std. Error	Standardized Coefficients: Beta	t	Sig.
1	(Constant)	-.493	.431		-1.144	.253
	Gender	.036	.036	.030	1.009	.313
	Ethni	.063	.049	.039	1.282	.200
	Age	.024	.015	.046	1.560	.119
	RL	.008	.037	.007	.224	.823
	GPtype2	.446	.049	.306	9.084	.000
	GPtype3	.529	.052	.350	10.264	.000
	GPtype4	.572	.055	.339	10.394	.000
	GPtype5	.815	.053	.503	15.311	.000

Coefficients[a]

Model		95.0% Confidence Interval for B Lower Bound	Upper Bound	Collinearity Statistics Tolerance	VIF
1	(Constant)	-1.340	.353		
	Gender	-.034	.106	.971	1.030
	Ethni	-.034	.160	.921	1.085
	Age	-.006	.054	.989	1.011
	RL	-.064	.080	.937	1.067
	GPtype2	.350	.543	.765	1.308
	GPtype3	.428	.630	.746	1.341
	GPtype4	.464	.681	.815	1.227
	GPtype5	.710	.919	.806	1.241

a. Dependent Variable: Promo

Collinearity Diagnostics[a]

Model	Dimension	Eigenvalue	Condition Index	Variance Proportions (Constant)	Gender	Ethni	Age
1	1	5.524	1.000	.00	.00	.00	.00
	2	1.004	2.346	.00	.00	.00	.00
	3	1.001	2.349	.00	.00	.00	.00
	4	1.000	2.350	.00	.00	.00	.00
	5	.301	4.285	.00	.03	.00	.00
	6	.093	7.724	.00	.78	.02	.00
	7	.055	10.036	.00	.13	.11	.00
	8	.023	15.614	.01	.06	.87	.02
	9	.001	82.224	.98	.00	.00	.98

Collinearity Diagnostics[a]

Model	Dimension	Variance Proportions RL	GPtype2	GPtype3	GPtype4	GPtype5
1	1	.00	.00	.00	.00	.00
	2	.00	.10	.43	.01	.08
	3	.00	.32	.01	.19	.13
	4	.00	.01	.01	.36	.31
	5	.00	.51	.44	.43	.46
	6	.19	.03	.06	.01	.01
	7	.80	.03	.00	.00	.01
	8	.00	.00	.05	.01	.00
	9	.00	.00	.00	.00	.00

a. Dependent Variable: Promo
