# Supplementary material for: Does a degree in medicine or a specialist programme or socioeconomic status advance the career of general practitioners in primary healthcare?
Source: PLoS One. 2026 Mar 30;21(3):e0346026. doi: 10.1371/journal.pone.0346026 (PMC13035118; doi:10.1371/journal.pone.0346026)
Supplement: S2_File — (PDF) [file pone.0346026.s002.pdf]

Between-Subjects Factors

		Value Label	N
GPtype	1.00	Full GP	285
	2.00	IM SPGP	170
	3.00	OB SPGP	155
	4.00	EM SPGP	118
	5.00	P SPGP	130
Gender	1.00	female	519
	2.00	male	339
Ethni	1.00	ethic	133
	2.00	han	725
RL	1.00	rural	320
	2.00	urban	538

Descriptive Statistics

Dependent Variable: CA

GPtype	Gender	Ethni	RL	Mean	Std. Deviation	N
Full GP	female	ethic	rural	1.0000	.79057	17
			urban	.8889	1.16667	9
			Total	.9615	.91568	26
		han	rural	1.2667	.97192	60
			urban	1.2206	.99018	68
			Total	1.2422	.97808	128
		Total	rural	1.2078	.93665	77
			urban	1.1818	1.00952	77
			Total	1.1948	.97067	154
	male	ethic	rural	1.6250	1.18773	8
			urban	.6667	.49237	12
			Total	1.0500	.94451	20
		han	rural	1.1143	.93215	35
			urban	1.0789	.93471	76
			Total	1.0901	.92980	111
		Total	rural	1.2093	.98942	43
			urban	1.0227	.89670	88
			Total	1.0840	.92851	131
	Total	ethic	rural	1.2000	.95743	25
			urban	.7619	.83095	21
			Total	1.0000	.91894	46
		han	rural	1.2105	.95533	95
			urban	1.1458	.96054	144
			Total	1.1715	.95699	239
		Total	rural	1.2083	.95174	120
			urban	1.0970	.95141	165
			Total	1.1439	.95147	285

Descriptive Statistics

Dependent Variable: CA

GPtype	Gender	Ethni	RL	Mean	Std. Deviation	N
IM SPGP	female	ethic	rural	1.0000	.00000	2
			urban	2.5000	.57735	4
			Total	2.0000	.89443	6
		han	rural	1.3556	1.28197	45
			urban	1.9355	1.15760	62
			Total	1.6916	1.23938	107
		Total	rural	1.3404	1.25588	47
			urban	1.9697	1.13639	66
			Total	1.7080	1.22242	113
	male	ethic	rural	1.5000	2.12132	2
			urban	2.0000	.	1
			Total	1.6667	1.52753	3
		han	rural	2.0400	.88882	25
			urban	1.9655	.82301	29
			Total	2.0000	.84675	54
		Total	rural	2.0000	.96077	27
			urban	1.9667	.80872	30
			Total	1.9825	.87610	57
	Total	ethic	rural	1.2500	1.25831	4
			urban	2.4000	.54772	5
			Total	1.8889	1.05409	9
		han	rural	1.6000	1.19661	70
			urban	1.9451	1.05791	91
			Total	1.7950	1.12981	161
		Total	rural	1.5811	1.19367	74
			urban	1.9687	1.04078	96
			Total	1.8000	1.12318	170
OB SPGP	female	ethic	rural	1.4706	.62426	17
			urban	1.6250	.61914	16
			Total	1.5455	.61699	33
		han	rural	1.5000	.52705	10
			urban	1.9306	.79304	72
			Total	1.8780	.77603	82
		Total	rural	1.4815	.57981	27
			urban	1.8750	.77032	88
			Total	1.7826	.74672	115
	male	ethic	rural	1.2857	.75593	7
			urban	2.0000	1.00000	3
			Total	1.5000	.84984	10
		han	rural	1.3333	.57735	3
			urban	1.9259	.67516	27
			Total	1.8667	.68145	30

Descriptive Statistics

Dependent Variable: CA

GPtype	Gender	Ethni	RL	Mean	Std. Deviation	N
		Total	rural	1.3000	.67495	10
			urban	1.9333	.69149	30
			Total	1.7750	.73336	40
	Total	ethic	rural	1.4167	.65386	24
			urban	1.6842	.67104	19
			Total	1.5349	.66722	43
		han	rural	1.4615	.51887	13
			urban	1.9293	.75933	99
			Total	1.8750	.74887	112
		Total	rural	1.4324	.60280	37
			urban	1.8898	.74860	118
			Total	1.7806	.74093	155
EM SPGP	female	ethic	rural	1.7143	.75593	7
			urban	1.3333	.57735	3
			Total	1.6000	.69921	10
		han	rural	1.7692	1.01274	13
			urban	1.9302	.79867	43
			Total	1.8929	.84592	56
		Total	rural	1.7500	.91047	20
			urban	1.8913	.79522	46
			Total	1.8485	.82727	66
	male	ethic	rural	2.1429	.89974	7
			urban	2.0000	1.41421	2
			Total	2.1111	.92796	9
		han	rural	2.1667	.71774	12
			urban	2.2903	.86385	31
			Total	2.2558	.81920	43
		Total	rural	2.1579	.76472	19
			urban	2.2727	.87581	33
			Total	2.2308	.83114	52
	Total	ethic	rural	1.9286	.82874	14
			urban	1.6000	.89443	5
			Total	1.8421	.83421	19
		han	rural	1.9600	.88882	25
			urban	2.0811	.84004	74
			Total	2.0505	.84965	99
		Total	rural	1.9487	.85682	39
			urban	2.0506	.84578	79
			Total	2.0169	.84715	118
P SPGP	female	ethic	rural	1.6000	.89443	5
			urban	2.3333	.57735	3
			Total	1.8750	.83452	8

Descriptive Statistics

Dependent Variable: CA

GPtype	Gender	Ethni	RL	Mean	Std. Deviation	N
		han	rural	2.0000	.72232	24
			urban	2.0513	.79302	39
			Total	2.0317	.76133	63
		Total	rural	1.9310	.75266	29
			urban	2.0714	.77752	42
			Total	2.0141	.76519	71
	male	ethic	rural	2.3333	.57735	3
			urban	2.2000	.44721	5
			Total	2.2500	.46291	8
		han	rural	2.0000	.68599	18
			urban	2.0000	.75000	33
			Total	2.0000	.72111	51
		Total	rural	2.0476	.66904	21
			urban	2.0263	.71610	38
			Total	2.0339	.69397	59
	Total	ethic	rural	1.8750	.83452	8
			urban	2.2500	.46291	8
			Total	2.0625	.68007	16
		han	rural	2.0000	.69843	42
			urban	2.0278	.76861	72
			Total	2.0175	.74051	114
		Total	rural	1.9800	.71400	50
			urban	2.0500	.74460	80
			Total	2.0231	.73099	130
Total	female	ethic	rural	1.3333	.75324	48
			urban	1.5714	.91670	35
			Total	1.4337	.82923	83
		han	rural	1.4671	1.04797	152
			urban	1.7782	.97861	284
			Total	1.6697	1.01306	436
		Total	rural	1.4350	.98520	200
			urban	1.7555	.97279	319
			Total	1.6320	.98904	519
	male	ethic	rural	1.7407	1.02254	27
			urban	1.3478	.93462	23
			Total	1.5600	.99304	50
		han	rural	1.6774	.94594	93
			urban	1.6735	.96901	196
			Total	1.6747	.96001	289
		Total	rural	1.6917	.95966	120
			urban	1.6393	.96855	219
			Total	1.6578	.96431	339

Descriptive Statistics

Dependent Variable: CA

GPtype	Gender	Ethni	RL	Mean	Std. Deviation	N
	Total	ethic	rural	1.4800	.87549	75
			urban	1.4828	.92227	58
			Total	1.4812	.89274	133
		han	rural	1.5469	1.01366	245
			urban	1.7354	.97505	480
			Total	1.6717	.99159	725
		Total	rural	1.5313	.98211	320
			urban	1.7082	.97184	538
			Total	1.6422	.97886	858

Levene's Test of Equality of Error Variances[a]

Dependent Variable: CA

F	df1	df2	Sig.
2.352	39	818	.000

Tests the null hypothesis that the error variance of the dependent variable is equal across groups.

a. Design: Intercept + Age + Gender + Ethni + RL + GPtype

Tests of Between-Subjects Effects

Dependent Variable: CA

Source	Type III Sum of Squares	df	Mean Square	F	Sig.	Partial Eta Squared
Corrected Model	121.058[a]	8	15.132	18.351	.000	.147
Intercept	.926	1	.926	1.123	.290	.001
Age	1.096	1	1.096	1.329	.249	.002
Gender	.780	1	.780	.946	.331	.001
Ethni	2.064	1	2.064	2.504	.114	.003
RL	2.212	1	2.212	2.682	.102	.003
GPtype	109.799	4	27.450	33.288	.000	.136
Error	700.095	849	.825			
Total	3135.000	858				
Corrected Total	821.153	857				

a. R Squared = .147 (Adjusted R Squared = .139)

Parameter Estimates

Dependent Variable: CA

Parameter	B	Std. Error	t	Sig.	95% Confidence Interval	
					Lower Bound	Upper Bound
Intercept	1.229	.777	1.581	.114	-.297	2.754
Age	.032	.028	1.153	.249	-.023	.087
[Gender=1.00]	-.063	.064	-.973	.331	-.189	.064
[Gender=2.00]	0[a]	.	.	.	.	.
[Ethni=1.00]	-.141	.089	-1.582	.114	-.316	.034
[Ethni=2.00]	0[a]	.	.	.	.	.
[RL=1.00]	-.108	.066	-1.638	.102	-.238	.022
[RL=2.00]	0[a]	.	.	.	.	.
[GPtype=1.00]	-.864	.096	-8.972	.000	-1.053	-.675
[GPtype=2.00]	-.211	.107	-1.983	.048	-.421	-.002
[GPtype=3.00]	-.220	.110	-1.990	.047	-.436	-.003
[GPtype=4.00]	-.005	.116	-.041	.967	-.232	.222
[GPtype=5.00]	0[a]	.	.	.	.	.

Parameter Estimates

Dependent Variable: CA

Parameter	Partial Eta Squared
Intercept	.003
Age	.002
[Gender=1.00]	.001
[Gender=2.00]	.
[Ethni=1.00]	.003
[Ethni=2.00]	.
[RL=1.00]	.003
[RL=2.00]	.
[GPtype=1.00]	.087
[GPtype=2.00]	.005
[GPtype=3.00]	.005
[GPtype=4.00]	.000
[GPtype=5.00]	.

a. This parameter is set to zero because it is redundant.

Estimated Marginal Means

GPtype

Estimates

Dependent Variable: CA

GPtype	Mean	Std. Error	95% Confidence Interval	
			Lower Bound	Upper Bound
Full GP	1.091[a]	.062	.971	1.212
IM SPGP	1.744[a]	.081	1.586	1.903
OB SPGP	1.736[a]	.078	1.582	1.890
EM SPGP	1.951[a]	.089	1.776	2.125
P SPGP	1.956[a]	.086	1.786	2.125

a. Covariates appearing in the model are evaluated at the following values: Age = 27.3695.

Pairwise Comparisons

Dependent Variable: CA

(I) GPtype	(J) GPtype	Mean Difference (I-J)	Std. Error	Sig.[b]	95% Confidence Interval for Difference[b]	
					Lower Bound	Upper Bound
Full GP	IM SPGP	-.653*	.089	.000	-.827	-.478
	OB SPGP	-.644*	.093	.000	-.828	-.461
	EM SPGP	-.859*	.100	.000	-1.055	-.664
	P SPGP	-.864*	.096	.000	-1.053	-.675
IM SPGP	Full GP	.653*	.089	.000	.478	.827
	OB SPGP	.008	.104	.937	-.197	.213
	EM SPGP	-.207	.110	.061	-.422	.009
	P SPGP	-.211*	.107	.048	-.421	-.002
OB SPGP	Full GP	.644*	.093	.000	.461	.828
	IM SPGP	-.008	.104	.937	-.213	.197
	EM SPGP	-.215	.112	.056	-.435	.006
	P SPGP	-.220*	.110	.047	-.436	-.003
EM SPGP	Full GP	.859*	.100	.000	.664	1.055
	IM SPGP	.207	.110	.061	-.009	.422
	OB SPGP	.215	.112	.056	-.006	.435
	P SPGP	-.005	.116	.967	-.232	.222
P SPGP	Full GP	.864*	.096	.000	.675	1.053
	IM SPGP	.211*	.107	.048	.002	.421
	OB SPGP	.220*	.110	.047	.003	.436
	EM SPGP	.005	.116	.967	-.222	.232

Based on estimated marginal means

*. The mean difference is significant at the .05 level.

b. Adjustment for multiple comparisons: Least Significant Difference (equivalent to no adjustments).

Univariate Tests

Dependent Variable: CA

	Sum of Squares	df	Mean Square	F	Sig.	Partial Eta Squared
Contrast	109.799	4	27.450	33.288	.000	.136
Error	700.095	849	.825			

The F tests the effect of GPtype. This test is based on the linearly independent pairwise comparisons among the estimated marginal means.
