# Supplementary material for: Does a degree in medicine or a specialist programme or socioeconomic status advance the career of general practitioners in primary healthcare?
Source: PLoS One. 2026 Mar 30;21(3):e0346026. doi: 10.1371/journal.pone.0346026 (PMC13035118; doi:10.1371/journal.pone.0346026)
Supplement: S3_ File — (PDF) [file pone.0346026.s003.pdf]

```
Mplus VERSION 8.3
MUTHEN & MUTHEN


INPUT INSTRUCTIONS

  DATA:
    FILE IS C:\Users\Sharon\Desktop\MydataVARIABLE:

    NAMES ARE Gender Ethni Age RL SES CA GPtype2
    GPtype3 GPtype4 GPtype5;

  USEVARIABLES ARE Gender Ethni Age RL SES CA GPType2 GPType3 GPType4 GPType5;
      MISSING ARE ALL (-9999);

    ANALYSIS:
      ESTIMATOR = ML;
      BOOTSTRAP = 5000;

    MODEL:
      SES ON GPType2 GPType3 GPType4 GPType5
             Gender Ethni Age RL;
      CA  ON SES GPType2 GPType3 GPType4 GPType5
             Gender Ethni Age RL;

    MODEL INDIRECT:
      CA IND GPType2;
      CA IND GPType3;
      CA IND GPType4;
      CA IND GPType5;

    OUTPUT:
      CINTERVAL(BCBOOTSTRAP);
      STANDARDIZED;



INPUT READING TERMINATED NORMALLY




SUMMARY OF ANALYSIS

Number of groups                                                 1
Number of observations                                         858

Number of dependent variables                                    2
Number of independent variables                                  8
Number of continuous latent variables                            0

Observed dependent variables

  Continuous
   SES         CA

Observed independent variables
   GENDER      ETHNI       AGE         RL          GPTYPE2     GPTYPE3
   GPTYPE4     GPTYPE5


Estimator                                                       ML
Information matrix                                        OBSERVED
```

```
Maximum number of iterations                                 1000
Convergence criterion                                    0.500D-04
Maximum number of steepest descent iterations                  20
Maximum number of iterations for H1                          2000
Convergence criterion for H1                             0.100D-03
Number of bootstrap draws
    Requested                                                5000
    Completed                                                5000

Input data file(s)
  C:\Users\Sharon\Desktop\Mydata\dat

Input data format  FREE
```

SUMMARY OF DATA

Number of missing data patterns 1

COVARIANCE COVERAGE OF DATA

Minimum covariance coverage value 0.100

PROPORTION OF DATA PRESENT

Covariance Coverage

	SES	CA	GENDER	ETHNI	AGE
SES	1.000				
CA	1.000	1.000			
GENDER	1.000	1.000	1.000		
ETHNI	1.000	1.000	1.000	1.000	
AGE	1.000	1.000	1.000	1.000	1.000
RL	1.000	1.000	1.000	1.000	1.000
GPTYPE2	1.000	1.000	1.000	1.000	1.000
GPTYPE3	1.000	1.000	1.000	1.000	1.000
GPTYPE4	1.000	1.000	1.000	1.000	1.000
GPTYPE5	1.000	1.000	1.000	1.000	1.000

Covariance Coverage

	RL	GPTYPE2	GPTYPE3	GPTYPE4	GPTYPE5
RL	1.000				
GPTYPE2	1.000	1.000			
GPTYPE3	1.000	1.000	1.000		
GPTYPE4	1.000	1.000	1.000	1.000	
GPTYPE5	1.000	1.000	1.000	1.000	1.000

UNIVARIATE SAMPLE STATISTICS

UNIVARIATE HIGHER-ORDER MOMENT DESCRIPTIVE STATISTICS

Variable/ Sample Size	Mean/ Variance	Skewness/ Kurtosis	Minimum/ Maximum	% with Min/Max	Percentiles 20%/60%	40%/80%	Median
SES	1.501	0.277	0.000	7.93%	1.000	1.000	1.000

	858.000	0.670	-0.523	3.000	13.05%	2.000	2.000	
CA		1.642	-0.095	0.000	13.40%	1.000	1.000	2.000
	858.000	0.957	-1.019	3.000	22.96%	2.000	3.000	
GENDER		1.395	0.429	1.000	60.49%	1.000	1.000	1.000
	858.000	0.239	-1.816	2.000	39.51%	1.000	2.000	
ETHNI		1.845	-1.906	1.000	15.50%	2.000	2.000	2.000
	858.000	0.131	1.635	2.000	84.50%	2.000	2.000	
AGE		27.369	0.547	25.000	0.12%	26.000	27.000	27.000
	858.000	1.240	-0.111	30.000	6.53%	28.000	28.000	
RL		1.627	-0.525	1.000	37.30%	1.000	2.000	2.000
	858.000	0.234	-1.724	2.000	62.70%	2.000	2.000	
GPTYPE2		2.578	0.402	1.000	33.22%	1.000	2.000	2.000
	858.000	2.092	-1.204	5.000	15.15%	3.000	4.000	
GPTYPE3		0.733	0.115	0.000	33.80%	0.000	1.000	1.000
	858.000	0.338	-0.514	2.000	7.11%	1.000	1.000	
GPTYPE4		0.528	0.253	0.000	49.07%	0.000	0.000	1.000
	858.000	0.287	-1.172	2.000	1.86%	1.000	1.000	
GPTYPE5		0.198	1.515	0.000	80.19%	0.000	0.000	0.000
	858.000	0.159	0.294	1.000	19.81%	0.000	0.000	

THE MODEL ESTIMATION TERMINATED NORMALLY

MODEL FIT INFORMATION

Number of Free Parameters 21

Loglikelihood

H0 Value -1455.328
H1 Value -1455.328

Information Criteria

Akaike (AIC) 2952.657
Bayesian (BIC) 3052.504
Sample-Size Adjusted BIC 2985.813
(n* = (n + 2) / 24)

Chi-Square Test of Model Fit

Value 0.000
Degrees of Freedom 0
P-Value 0.0000

RMSEA (Root Mean Square Error Of Approximation)

Estimate 0.000
90 Percent C.I. 0.000 0.000
Probability RMSEA <= .05 0.000

CFI/TLI

CFI 1.000
TLI 1.000

Chi-Square Test of Model Fit for the Baseline Model

Value 1577.329
Degrees of Freedom 17
P-Value 0.0000

SRMR (Standardized Root Mean Square Residual)

Value	0.000

MODEL RESULTS

	Estimate	S.E.	Est./S.E.	Two-Tailed P-Value
SES ON				
GPTYPE2	0.204	0.019	10.606	0.000
GPTYPE3	0.331	0.043	7.647	0.000
GPTYPE4	0.284	0.046	6.178	0.000
GPTYPE5	0.450	0.059	7.565	0.000
GENDER	-0.039	0.048	-0.805	0.421
ETHNI	0.004	0.063	0.057	0.955
AGE	-0.005	0.022	-0.222	0.825
RL	0.203	0.049	4.162	0.000
CA ON				
SES	0.133	0.027	4.957	0.000
GPTYPE2	0.031	0.015	2.061	0.039
GPTYPE3	1.163	0.039	29.473	0.000
GPTYPE4	1.032	0.030	34.696	0.000
GPTYPE5	0.293	0.051	5.796	0.000
GENDER	0.016	0.033	0.476	0.634
ETHNI	0.057	0.041	1.375	0.169
AGE	0.010	0.014	0.691	0.490
RL	0.085	0.033	2.542	0.011
Intercepts				
SES	0.349	0.618	0.564	0.573
CA	-0.624	0.398	-1.567	0.117
Residual Variances				
SES	0.471	0.020	23.849	0.000
CA	0.217	0.013	16.607	0.000

STANDARDIZED MODEL RESULTS

STDYX Standardization

	Estimate	S.E.	Est./S.E.	Two-Tailed P-Value
SES ON				
GPTYPE2	0.360	0.031	11.789	0.000
GPTYPE3	0.235	0.030	7.777	0.000
GPTYPE4	0.186	0.029	6.310	0.000
GPTYPE5	0.219	0.029	7.498	0.000
GENDER	-0.023	0.029	-0.805	0.421
ETHNI	0.002	0.028	0.057	0.955
AGE	-0.007	0.031	-0.222	0.825
RL	0.120	0.028	4.202	0.000
CA ON				
SES	0.111	0.023	4.895	0.000
GPTYPE2	0.046	0.022	2.067	0.039

	Estimate	S.E.	Est./S.E.	Two-Tailed P-Value
GPTYPE3	0.691	0.020	34.668	0.000
GPTYPE4	0.565	0.015	37.526	0.000
GPTYPE5	0.119	0.021	5.595	0.000
GENDER	0.008	0.016	0.476	0.634
ETHNI	0.021	0.015	1.375	0.169
AGE	0.011	0.016	0.691	0.489
RL	0.042	0.017	2.543	0.011
**Intercepts**				
SES	0.426	0.756	0.564	0.573
CA	-0.638	0.407	-1.565	0.118
**Residual Variances**				
SES	0.703	0.024	28.733	0.000
CA	0.226	0.016	14.231	0.000

STDY Standardization

	Estimate	S.E.	Est./S.E.	Two-Tailed P-Value
**SES ON**				
GPTYPE2	0.249	0.022	11.488	0.000
GPTYPE3	0.404	0.052	7.797	0.000
GPTYPE4	0.347	0.056	6.245	0.000
GPTYPE5	0.549	0.072	7.656	0.000
GENDER	-0.048	0.059	-0.805	0.421
ETHNI	0.004	0.076	0.057	0.955
AGE	-0.006	0.027	-0.221	0.825
RL	0.248	0.059	4.207	0.000
**CA ON**				
SES	0.111	0.023	4.895	0.000
GPTYPE2	0.032	0.015	2.061	0.039
GPTYPE3	1.188	0.040	30.071	0.000
GPTYPE4	1.055	0.029	36.734	0.000
GPTYPE5	0.299	0.053	5.682	0.000
GENDER	0.016	0.033	0.476	0.634
ETHNI	0.058	0.042	1.372	0.170
AGE	0.010	0.014	0.690	0.490
RL	0.087	0.034	2.544	0.011
**Intercepts**				
SES	0.426	0.756	0.564	0.573
CA	-0.638	0.407	-1.565	0.118
**Residual Variances**				
SES	0.703	0.024	28.733	0.000
CA	0.226	0.016	14.231	0.000

STD Standardization

	Estimate	S.E.	Est./S.E.	Two-Tailed P-Value
**SES ON**				
GPTYPE2	0.204	0.019	10.606	0.000
GPTYPE3	0.331	0.043	7.647	0.000
GPTYPE4	0.284	0.046	6.178	0.000
GPTYPE5	0.450	0.059	7.565	0.000
GENDER	-0.039	0.048	-0.805	0.421

```
    ETHNI              0.004      0.063      0.057      0.955
    AGE               -0.005      0.022     -0.222      0.825
    RL                 0.203      0.049      4.162      0.000

 CA       ON
    SES                0.133      0.027      4.957      0.000
    GPTYPE2            0.031      0.015      2.061      0.039
    GPTYPE3            1.163      0.039     29.473      0.000
    GPTYPE4            1.032      0.030     34.696      0.000
    GPTYPE5            0.293      0.051      5.796      0.000
    GENDER             0.016      0.033      0.476      0.634
    ETHNI              0.057      0.041      1.375      0.169
    AGE                0.010      0.014      0.691      0.490
    RL                 0.085      0.033      2.542      0.011

 Intercepts
    SES                0.349      0.618      0.564      0.573
    CA                -0.624      0.398     -1.567      0.117

 Residual Variances
    SES                0.471      0.020     23.849      0.000
    CA                 0.217      0.013     16.607      0.000


R-SQUARE

    Observed                                        Two-Tailed
    Variable        Estimate       S.E.  Est./S.E.    P-Value

    SES                0.297      0.024     12.157      0.000
    CA                 0.774      0.016     48.632      0.000


TOTAL, TOTAL INDIRECT, SPECIFIC INDIRECT, AND DIRECT EFFECTS


                                                    Two-Tailed
                    Estimate       S.E.  Est./S.E.    P-Value

Effects from GPTYPE2 to CA

  Total                0.058      0.014      4.224      0.000
  Total indirect       0.027      0.006      4.528      0.000

  Specific indirect 1
    CA
    SES
    GPTYPE2            0.027      0.006      4.528      0.000

  Direct
    CA
    GPTYPE2            0.031      0.015      2.061      0.039


Effects from GPTYPE3 to CA

  Total                1.207      0.038     31.897      0.000
  Total indirect       0.044      0.011      4.036      0.000

  Specific indirect 1
    CA
    SES
    GPTYPE3            0.044      0.011      4.036      0.000
```

```
  Direct
    CA
    GPTYPE3            1.163      0.039     29.473      0.000


Effects from GPTYPE4 to CA

  Total              1.069      0.030     35.731      0.000
  Total indirect     0.038      0.010      3.820      0.000

  Specific indirect 1
    CA
    SES
    GPTYPE4            0.038      0.010      3.820      0.000

  Direct
    CA
    GPTYPE4            1.032      0.030     34.696      0.000


Effects from GPTYPE5 to CA

  Total              0.352      0.045      7.851      0.000
  Total indirect     0.060      0.014      4.243      0.000

  Specific indirect 1
    CA
    SES
    GPTYPE5            0.060      0.014      4.243      0.000

  Direct
    CA
    GPTYPE5            0.293      0.051      5.796      0.000



STANDARDIZED TOTAL, TOTAL INDIRECT, SPECIFIC INDIRECT, AND DIRECT EFFECTS


STDYX Standardization

                                                    Two-Tailed
                    Estimate       S.E.  Est./S.E.    P-Value

Effects from GPTYPE2 to CA

  Total              0.086      0.020      4.230      0.000
  Total indirect     0.040      0.009      4.487      0.000

  Specific indirect 1
    CA
    SES
    GPTYPE2            0.040      0.009      4.487      0.000

  Direct
    CA
    GPTYPE2            0.046      0.022      2.067      0.039


Effects from GPTYPE3 to CA

  Total              0.717      0.019     37.048      0.000
```

```
  Total indirect       0.026      0.007      3.988      0.000

  Specific indirect 1
    CA
    SES
    GPTYPE3            0.026      0.007      3.988      0.000

  Direct
    CA
    GPTYPE3            0.691      0.020     34.668      0.000


Effects from GPTYPE4 to CA

  Total                0.585      0.015     38.501      0.000
  Total indirect       0.021      0.005      3.817      0.000

  Specific indirect 1
    CA
    SES
    GPTYPE4            0.021      0.005      3.817      0.000

  Direct
    CA
    GPTYPE4            0.565      0.015     37.526      0.000


Effects from GPTYPE5 to CA

  Total                0.144      0.019      7.517      0.000
  Total indirect       0.024      0.006      4.326      0.000

  Specific indirect 1
    CA
    SES
    GPTYPE5            0.024      0.006      4.326      0.000

  Direct
    CA
    GPTYPE5            0.119      0.021      5.595      0.000



STDY Standardization

                                                        Two-Tailed
                    Estimate       S.E.  Est./S.E.    P-Value

Effects from GPTYPE2 to CA

  Total                0.059      0.014      4.225      0.000
  Total indirect       0.028      0.006      4.525      0.000

  Specific indirect 1
    CA
    SES
    GPTYPE2            0.028      0.006      4.525      0.000

  Direct
    CA
    GPTYPE2            0.032      0.015      2.061      0.039
```

```
Effects from GPTYPE3 to CA

  Total                1.233      0.039     31.864      0.000
  Total indirect       0.045      0.011      3.989      0.000

  Specific indirect 1
    CA
    SES
    GPTYPE3            0.045      0.011      3.989      0.000

  Direct
    CA
    GPTYPE3            1.188      0.040     30.071      0.000


Effects from GPTYPE4 to CA

  Total                1.093      0.029     37.211      0.000
  Total indirect       0.038      0.010      3.797      0.000

  Specific indirect 1
    CA
    SES
    GPTYPE4            0.038      0.010      3.797      0.000

  Direct
    CA
    GPTYPE4            1.055      0.029     36.734      0.000


Effects from GPTYPE5 to CA

  Total                0.360      0.047      7.631      0.000
  Total indirect       0.061      0.014      4.248      0.000

  Specific indirect 1
    CA
    SES
    GPTYPE5            0.061      0.014      4.248      0.000

  Direct
    CA
    GPTYPE5            0.299      0.053      5.682      0.000



STD Standardization

                                                        Two-Tailed
                    Estimate       S.E.  Est./S.E.    P-Value

Effects from GPTYPE2 to CA

  Total                0.058      0.014      4.224      0.000
  Total indirect       0.027      0.006      4.528      0.000

  Specific indirect 1
    CA
    SES
    GPTYPE2            0.027      0.006      4.528      0.000

  Direct
    CA
```

```
    GPTYPE2            0.031      0.015      2.061      0.039


Effects from GPTYPE3 to CA

  Total                1.207      0.038     31.897      0.000
  Total indirect       0.044      0.011      4.036      0.000

  Specific indirect 1
    CA
    SES
    GPTYPE3            0.044      0.011      4.036      0.000

  Direct
    CA
    GPTYPE3            1.163      0.039     29.473      0.000


Effects from GPTYPE4 to CA

  Total                1.069      0.030     35.731      0.000
  Total indirect       0.038      0.010      3.820      0.000

  Specific indirect 1
    CA
    SES
    GPTYPE4            0.038      0.010      3.820      0.000

  Direct
    CA
    GPTYPE4            1.032      0.030     34.696      0.000


Effects from GPTYPE5 to CA

  Total                0.352      0.045      7.851      0.000
  Total indirect       0.060      0.014      4.243      0.000

  Specific indirect 1
    CA
    SES
    GPTYPE5            0.060      0.014      4.243      0.000

  Direct
    CA
    GPTYPE5            0.293      0.051      5.796      0.000



CONFIDENCE INTERVALS OF MODEL RESULTS

                  Lower .5%  Lower 2.5%    Lower 5%    Estimate    Upper 5%  Upper 2.5%   Upper .5%

 SES      ON
    GPTYPE2          0.153       0.165       0.172       0.204       0.234       0.241       0.252
    GPTYPE3          0.224       0.251       0.262       0.331       0.404       0.419       0.446
    GPTYPE4          0.166       0.196       0.208       0.284       0.360       0.374       0.403
    GPTYPE5          0.298       0.338       0.355       0.450       0.551       0.567       0.597
    GENDER          -0.155      -0.133      -0.118      -0.039       0.042       0.058       0.087
    ETHNI           -0.159      -0.119      -0.098       0.004       0.106       0.127       0.161
    AGE             -0.062      -0.048      -0.041      -0.005       0.033       0.040       0.054
    RL               0.076       0.103       0.119       0.203       0.279       0.295       0.330
```

CA ON							
SES	0.061	0.078	0.087	0.133	0.175	0.184	0.201
GPTYPE2	-0.006	0.002	0.006	0.031	0.056	0.060	0.070
GPTYPE3	1.062	1.086	1.099	1.163	1.227	1.239	1.269
GPTYPE4	0.955	0.974	0.984	1.032	1.082	1.090	1.109
GPTYPE5	0.166	0.194	0.209	0.293	0.375	0.392	0.420
GENDER	-0.066	-0.048	-0.037	0.016	0.070	0.080	0.099
ETHNI	-0.049	-0.025	-0.012	0.057	0.124	0.137	0.165
AGE	-0.027	-0.018	-0.014	0.010	0.032	0.037	0.044
RL	0.003	0.021	0.031	0.085	0.142	0.152	0.173
Intercepts							
SES	-1.266	-0.880	-0.661	0.349	1.368	1.556	1.910
CA	-1.630	-1.391	-1.269	-0.624	0.048	0.171	0.394
Residual Variances							
SES	0.426	0.437	0.443	0.471	0.508	0.512	0.523
CA	0.189	0.195	0.199	0.217	0.243	0.247	0.260

CONFIDENCE INTERVALS OF STANDARDIZED MODEL RESULTS

STDYX Standardization

	Lower .5%	Lower 2.5%	Lower 5%	Estimate	Upper 5%	Upper 2.5%	Upper .5%
SES ON							
GPTYPE2	0.278	0.298	0.308	0.360	0.409	0.418	0.436
GPTYPE3	0.159	0.177	0.186	0.235	0.285	0.294	0.314
GPTYPE4	0.110	0.129	0.137	0.186	0.235	0.243	0.261
GPTYPE5	0.145	0.163	0.172	0.219	0.267	0.278	0.291
GENDER	-0.093	-0.080	-0.070	-0.023	0.025	0.034	0.052
ETHNI	-0.071	-0.053	-0.043	0.002	0.047	0.056	0.073
AGE	-0.085	-0.065	-0.057	-0.007	0.045	0.054	0.074
RL	0.046	0.062	0.071	0.120	0.165	0.173	0.193
CA ON							
SES	0.052	0.066	0.072	0.111	0.148	0.155	0.169
GPTYPE2	-0.010	0.002	0.009	0.046	0.082	0.088	0.103
GPTYPE3	0.638	0.651	0.658	0.691	0.723	0.729	0.741
GPTYPE4	0.525	0.534	0.540	0.565	0.589	0.593	0.602
GPTYPE5	0.067	0.079	0.085	0.119	0.155	0.162	0.176
GENDER	-0.034	-0.024	-0.019	0.008	0.035	0.041	0.049
ETHNI	-0.018	-0.009	-0.004	0.021	0.046	0.051	0.061
AGE	-0.031	-0.021	-0.016	0.011	0.037	0.042	0.051
RL	0.002	0.011	0.016	0.042	0.070	0.075	0.085
Intercepts							
SES	-1.536	-1.077	-0.801	0.426	1.668	1.917	2.352
CA	-1.640	-1.428	-1.287	-0.638	0.049	0.173	0.398
Residual Variances							
SES	0.645	0.660	0.669	0.703	0.749	0.754	0.768
CA	0.191	0.200	0.205	0.226	0.259	0.264	0.273

STDY Standardization

	Lower .5%	Lower 2.5%	Lower 5%	Estimate	Upper 5%	Upper 2.5%	Upper .5%
SES ON							

GPTYPE2	0.190	0.204	0.212	0.249	0.283	0.290	0.302
GPTYPE3	0.273	0.307	0.322	0.404	0.491	0.507	0.543
GPTYPE4	0.206	0.240	0.255	0.347	0.438	0.456	0.492
GPTYPE5	0.370	0.414	0.434	0.549	0.670	0.692	0.725
GENDER	-0.190	-0.164	-0.144	-0.048	0.051	0.070	0.105
ETHNI	-0.198	-0.146	-0.119	0.004	0.130	0.155	0.198
AGE	-0.075	-0.058	-0.051	-0.006	0.040	0.048	0.066
RL	0.095	0.127	0.146	0.248	0.340	0.358	0.399
**CA ON**							
SES	0.052	0.066	0.072	0.111	0.148	0.155	0.169
GPTYPE2	-0.007	0.002	0.006	0.032	0.057	0.061	0.072
GPTYPE3	1.086	1.112	1.125	1.188	1.253	1.267	1.294
GPTYPE4	0.979	0.998	1.008	1.055	1.101	1.111	1.130
GPTYPE5	0.170	0.198	0.213	0.299	0.387	0.402	0.438
GENDER	-0.068	-0.049	-0.038	0.016	0.072	0.083	0.101
ETHNI	-0.051	-0.026	-0.012	0.058	0.126	0.140	0.168
AGE	-0.028	-0.019	-0.015	0.010	0.033	0.038	0.045
RL	0.003	0.022	0.032	0.087	0.145	0.155	0.176
**Intercepts**							
SES	-1.536	-1.077	-0.801	0.426	1.668	1.917	2.352
CA	-1.640	-1.428	-1.287	-0.638	0.049	0.173	0.398
**Residual Variances**							
SES	0.645	0.660	0.669	0.703	0.749	0.754	0.768
CA	0.191	0.200	0.205	0.226	0.259	0.264	0.273

STD Standardization

	Lower .5%	Lower 2.5%	Lower 5%	Estimate	Upper 5%	Upper 2.5%	Upper .5%
**SES ON**							
GPTYPE2	0.153	0.165	0.172	0.204	0.234	0.241	0.252
GPTYPE3	0.224	0.251	0.262	0.331	0.404	0.419	0.446
GPTYPE4	0.166	0.196	0.208	0.284	0.360	0.374	0.403
GPTYPE5	0.298	0.338	0.355	0.450	0.551	0.567	0.597
GENDER	-0.155	-0.133	-0.118	-0.039	0.042	0.058	0.087
ETHNI	-0.159	-0.119	-0.098	0.004	0.106	0.127	0.161
AGE	-0.062	-0.048	-0.041	-0.005	0.033	0.040	0.054
RL	0.076	0.103	0.119	0.203	0.279	0.295	0.330
**CA ON**							
SES	0.061	0.078	0.087	0.133	0.175	0.184	0.201
GPTYPE2	-0.006	0.002	0.006	0.031	0.056	0.060	0.070
GPTYPE3	1.062	1.086	1.099	1.163	1.227	1.239	1.269
GPTYPE4	0.955	0.974	0.984	1.032	1.082	1.090	1.109
GPTYPE5	0.166	0.194	0.209	0.293	0.375	0.392	0.420
GENDER	-0.066	-0.048	-0.037	0.016	0.070	0.080	0.099
ETHNI	-0.049	-0.025	-0.012	0.057	0.124	0.137	0.165
AGE	-0.027	-0.018	-0.014	0.010	0.032	0.037	0.044
RL	0.003	0.021	0.031	0.085	0.142	0.152	0.173
**Intercepts**							
SES	-1.266	-0.880	-0.661	0.349	1.368	1.556	1.910
CA	-1.630	-1.391	-1.269	-0.624	0.048	0.171	0.394
**Residual Variances**							
SES	0.426	0.437	0.443	0.471	0.508	0.512	0.523
CA	0.189	0.195	0.199	0.217	0.243	0.247	0.260

CONFIDENCE INTERVALS OF TOTAL, TOTAL INDIRECT, SPECIFIC INDIRECT, AND DIRECT EFFECTS

	Lower .5%	Lower 2.5%	Lower 5%	Estimate	Upper 5%	Upper 2.5%	Upper .5%
Effects from GPTYPE2 to CA							
Total	0.023	0.030	0.035	0.058	0.081	0.085	0.094
Total indirect	0.012	0.016	0.018	0.027	0.037	0.039	0.044
Specific indirect 1							
CA							
SES							
GPTYPE2	0.012	0.016	0.018	0.027	0.037	0.039	0.044
Direct							
CA							
GPTYPE2	-0.006	0.002	0.006	0.031	0.056	0.060	0.070
Effects from GPTYPE3 to CA							
Total	1.110	1.134	1.145	1.207	1.268	1.280	1.307
Total indirect	0.020	0.025	0.028	0.044	0.063	0.068	0.076
Specific indirect 1							
CA							
SES							
GPTYPE3	0.020	0.025	0.028	0.044	0.063	0.068	0.076
Direct							
CA							
GPTYPE3	1.062	1.086	1.099	1.163	1.227	1.239	1.269
Effects from GPTYPE4 to CA							
Total	0.991	1.010	1.020	1.069	1.119	1.128	1.145
Total indirect	0.016	0.021	0.023	0.038	0.056	0.060	0.067
Specific indirect 1							
CA							
SES							
GPTYPE4	0.016	0.021	0.023	0.038	0.056	0.060	0.067
Direct							
CA							
GPTYPE4	0.955	0.974	0.984	1.032	1.082	1.090	1.109
Effects from GPTYPE5 to CA							
Total	0.239	0.266	0.277	0.352	0.424	0.439	0.466
Total indirect	0.027	0.035	0.039	0.060	0.085	0.090	0.100
Specific indirect 1							
CA							
SES							
GPTYPE5	0.027	0.035	0.039	0.060	0.085	0.090	0.100
Direct							
CA							
GPTYPE5	0.166	0.194	0.209	0.293	0.375	0.392	0.420

CONFIDENCE INTERVALS OF STANDARDIZED TOTAL, TOTAL INDIRECT, SPECIFIC INDIRECT, AND DIRECT EFFECTS

STDYX Standardization

	Lower .5%	Lower 2.5%	Lower 5%	Estimate	Upper 5%	Upper 2.5%	Upper .5%
Effects from GPTYPE2 to CA							
Total	0.034	0.045	0.052	0.086	0.119	0.125	0.137
Total indirect	0.019	0.023	0.026	0.040	0.056	0.059	0.065
Specific indirect 1							
CA							
SES							
GPTYPE2	0.019	0.023	0.026	0.040	0.056	0.059	0.065
Direct							
CA							
GPTYPE2	-0.010	0.002	0.009	0.046	0.082	0.088	0.103
Effects from GPTYPE3 to CA							
Total	0.666	0.677	0.684	0.717	0.747	0.754	0.765
Total indirect	0.012	0.015	0.016	0.026	0.038	0.040	0.045
Specific indirect 1							
CA							
SES							
GPTYPE3	0.012	0.015	0.016	0.026	0.038	0.040	0.045
Direct							
CA							
GPTYPE3	0.638	0.651	0.658	0.691	0.723	0.729	0.741
Effects from GPTYPE4 to CA							
Total	0.545	0.556	0.560	0.585	0.610	0.615	0.624
Total indirect	0.009	0.011	0.013	0.021	0.031	0.033	0.037
Specific indirect 1							
CA							
SES							
GPTYPE4	0.009	0.011	0.013	0.021	0.031	0.033	0.037
Direct							
CA							
GPTYPE4	0.525	0.534	0.540	0.565	0.589	0.593	0.602
Effects from GPTYPE5 to CA							
Total	0.096	0.108	0.112	0.144	0.175	0.181	0.193
Total indirect	0.011	0.014	0.016	0.024	0.035	0.037	0.041
Specific indirect 1							
CA							
SES							

```
    GPTYPE5          0.011       0.014       0.016       0.024       0.035       0.037       0.041

  Direct
    CA
    GPTYPE5          0.067       0.079       0.085       0.119       0.155       0.162       0.176



STDY Standardization

                  Lower .5%  Lower 2.5%    Lower 5%    Estimate    Upper 5%  Upper 2.5%   Upper .5%

Effects from GPTYPE2 to CA

  Total              0.023       0.031       0.036       0.059       0.082       0.087       0.096
  Total indirect     0.013       0.016       0.018       0.028       0.038       0.040       0.045

  Specific indirect 1
    CA
    SES
    GPTYPE2          0.013       0.016       0.018       0.028       0.038       0.040       0.045

  Direct
    CA
    GPTYPE2         -0.007       0.002       0.006       0.032       0.057       0.061       0.072


Effects from GPTYPE3 to CA

  Total              1.138       1.158       1.170       1.233       1.295       1.308       1.335
  Total indirect     0.020       0.025       0.028       0.045       0.065       0.069       0.079

  Specific indirect 1
    CA
    SES
    GPTYPE3          0.020       0.025       0.028       0.045       0.065       0.069       0.079

  Direct
    CA
    GPTYPE3          1.086       1.112       1.125       1.188       1.253       1.267       1.294


Effects from GPTYPE4 to CA

  Total              1.012       1.034       1.044       1.093       1.139       1.148       1.167
  Total indirect     0.017       0.021       0.023       0.038       0.057       0.061       0.068

  Specific indirect 1
    CA
    SES
    GPTYPE4          0.017       0.021       0.023       0.038       0.057       0.061       0.068

  Direct
    CA
    GPTYPE4          0.979       0.998       1.008       1.055       1.101       1.111       1.130


Effects from GPTYPE5 to CA

  Total              0.242       0.269       0.282       0.360       0.437       0.451       0.484
  Total indirect     0.028       0.036       0.040       0.061       0.087       0.092       0.103

  Specific indirect 1
```

CA							
SES							
GPTYPE5	0.028	0.036	0.040	0.061	0.087	0.092	0.103
Direct							
CA							
GPTYPE5	0.170	0.198	0.213	0.299	0.387	0.402	0.438

STD Standardization

	Lower .5%	Lower 2.5%	Lower 5%	Estimate	Upper 5%	Upper 2.5%	Upper .5%
Effects from GPTYPE2 to CA							
Total	0.023	0.030	0.035	0.058	0.081	0.085	0.094
Total indirect	0.012	0.016	0.018	0.027	0.037	0.039	0.044
Specific indirect 1							
CA							
SES							
GPTYPE2	0.012	0.016	0.018	0.027	0.037	0.039	0.044
Direct							
CA							
GPTYPE2	-0.006	0.002	0.006	0.031	0.056	0.060	0.070
Effects from GPTYPE3 to CA							
Total	1.110	1.134	1.145	1.207	1.268	1.280	1.307
Total indirect	0.020	0.025	0.028	0.044	0.063	0.068	0.076
Specific indirect 1							
CA							
SES							
GPTYPE3	0.020	0.025	0.028	0.044	0.063	0.068	0.076
Direct							
CA							
GPTYPE3	1.062	1.086	1.099	1.163	1.227	1.239	1.269
Effects from GPTYPE4 to CA							
Total	0.991	1.010	1.020	1.069	1.119	1.128	1.145
Total indirect	0.016	0.021	0.023	0.038	0.056	0.060	0.067
Specific indirect 1							
CA							
SES							
GPTYPE4	0.016	0.021	0.023	0.038	0.056	0.060	0.067
Direct							
CA							
GPTYPE4	0.955	0.974	0.984	1.032	1.082	1.090	1.109
Effects from GPTYPE5 to CA							
Total	0.239	0.266	0.277	0.352	0.424	0.439	0.466
Total indirect	0.027	0.035	0.039	0.060	0.085	0.090	0.100

Specific indirect 1							
CA							
SES							
GPTYPE5	0.027	0.035	0.039	0.060	0.085	0.090	0.100
Direct							
CA							
GPTYPE5	0.166	0.194	0.209	0.293	0.375	0.392	0.420
